# Randomized, Negative-Controlled Pilot Study on the Treatment of Intramammary *Staphylococcus aureus* Infections in Dairy Cows with a Bacteriophage Cocktail

**DOI:** 10.3390/antibiotics15010032

**Published:** 2026-01-01

**Authors:** Volker Krömker, Stefanie Leimbach, Anne Tellen, Nicole Wente, Janina Schmidt, Hansjörg Lehnherr, Franziska Nankemann

**Affiliations:** 1 Department of Bioprocess Engineering and Microbiology, University of Applied Sciences and Arts, Hannover, D-30453 Hannover, Germany; 2 Phage Technology Center GmbH, D-59199 Bönen, Germany

**Keywords:** mastitis, intramammary infection, *S. aureus*, bacteriophages, therapy

## Abstract

**Background/Objectives**: *Staphylococcus (S.) aureus* is a major pathogen causing bovine mastitis and is often refractory to antibiotic therapies due to virulence factors and resistance mechanisms. In this pilot study, the safety and efficacy of an intramammary phage cocktail, in naturally *S. aureus*-infected dairy cows, were investigated. **Methods**: The initial part of the study on farm 1 confirmed tolerability and safety, as there were no observed systemic side effects of treatment. The subsequent efficacy study on farm 2 included 23 with *S. aureus* infected udder quarters, which were randomly divided into a treatment group (*n* = 16) and a control group (*n* = 7). The quarters in the treatment group received five intramammary infusions of the phage cocktail at 12-h intervals. **Results**: This resulted in a bacteriological cure rate of 81.3% (13/16) for the treatment group, compared to 28.6% (2/7) in the control group (*p* = 0.026). **Conclusions:** These results indicate that phage therapy is well-tolerated and may be a promising alternative to antibiotics for treating *S. aureus* mastitis, although confirmation in larger-scale, multicenter studies is required.

## 1. Introduction

*Staphylococcus aureus* is one of the most important pathogens causing bovine mastitis worldwide. In our own prevalence studies from Germany, it was shown that infections of mammary glands with *S. aureus* were detectable in 78% of farms. Animal prevalence rates of 3 to 10.7% were found in the affected herds [1]. *S. aureus* is a typical contagious pathogen primarily transmitted between cows during milking, with contaminated teat liners, milker’s hands, and milk serving as the main vectors of spread [2,3]. It is particularly associated with long-lasting intramammary infections and has the ability to persist, often without clinical signs [4,5]. *S. aureus* is able to express a wide range of virulence factors, such as the formation of biofilms or adherence [6,7]. Additionally, *S. aureus* could evade the host’s immune response by forming microabscesses and surviving intracellularly. *S. aureus* is also known for producing β-lactamases, leading to resistance against many commonly used antibiotics. Consequently, both spontaneous and antibiotic-induced cure rates for *S. aureus* mastitis are typically low, especially in cases of subclinical or chronic infection. In a recent study, the lowest bacteriological cure rate after antibiotic therapy was found for *S. aureus* (44.7%) in 2883 clinical cases of mastitis from Northern Germany [8]. Various studies have shown that the success of treatment depends on various factors, such as the age of the animal, the cell count of the milk of the infected quarter, or the age of the infection [9,10,11,12]. Very different recommendations are given for the treatment of subclinical *S. aureus* mastitis. While some authors recommend antibiotic therapy, and this is also regularly carried out on many dairy farms, other authors conclude that treatment of subclinical cases in lactation cannot be recommended due to insufficient cure rates and high costs [13,14].

Due to the limited success of conventional antibiotic therapies and increasing concerns about antimicrobial resistance, bacteriophages (phages) are gaining renewed attention as an alternative or adjunctive treatment approach, particularly for targeting antibiotic-resistant strains [15]. Bacteriophages are viruses that specifically infect and lyse bacteria and thus represent the natural predators of bacteria. They are ubiquitous in environments where bacterial populations exist [16]. The use of bacteriophages in veterinary medicine is generally regarded as safe for both treated animals and for humans consuming animal-derived products post-treatment. This safety assumption is supported by the fact that higher organisms, including humans, are continuously exposed to billions of bacteriophages in their natural environment without exhibiting adverse effects [17,18,19,20].

Previous *in vitro* studies of our working group have demonstrated the lytic activity of phages against *S. aureus* isolates from bovine mastitis cases [21]. However, thus far, only a few controlled *in vivo* studies assessing the efficacy and safety of bacteriophages have been reported, using murine mastitis models infected with bovine-associated *S. aureus* strains [22]. To date, the efficacy of phage therapy has only been tested in one clinical trial on dairy cows. The difference found with regard to the *S. aureus* cure rate was not significant. Regarding the effect of phage therapy on the somatic cell count, Gill et al. showed that a single infusion of the phage preparation to healthy udder quarters led to a strong increase in the somatic cell count in the milk [5].

This pilot study aimed to evaluate the tolerability, safety, and therapeutic potential of an intramammarily administered phage cocktail in dairy cows naturally infected with *S. aureus*. The trial was conducted in two commercial German dairy herds, using a randomized, negative-controlled design.

## 2. Results

### 2.1. Study Population and Enrollment

On Farm 1, five animals were pre-screened, of which three cows with confirmed *S. aureus* infection (one infected quarter per animal) were enrolled in the tolerability trial. On Farm 2, 72 animals were pre-screened, resulting in 18 animals with 23 *S. aureus*-infected udder quarters meeting the inclusion criteria and being enrolled in the efficacy trial.

### 2.2. Farm 1—Tolerance Trial

The three cows were successively treated with the phage solution in three udder quarters each (one infected quarter and two non-infected quarters per animal). The fourth udder quarter served as untreated control.

The animals showed no defensive reactions during application. No fever or other disorders of general condition were observed in the animals. Two animals developed slight flakes in the treated, *S. aureus*-infected udder quarters, but this was expected as a reaction to the phages. Thus, the tolerance of the phage solution could be demonstrated. This part of the study showed that the application of specific, effective bacteriophages to affected udder quarters (those infected with a sensitive *S. aureus* strain) can lead to short-term local reactions (change in electrical conductivity, formation of a few fine flakes, local tissue reactions). Healthy udder quarters did not show development of flakes after phage application.

### 2.3. Farm 2—Efficacy Trial

A total of 18 animals with 23 *S. aureus*-infected udder quarters were enrolled in the efficacy trial of the study based on the inclusion criteria described above. These udder quarters were randomly assigned to either the experimental or control group, resulting in 16 quarters in the experimental group and 7 quarters in the control group. The animals showed no defensive reactions during application. General disorders did not occur. The tolerance of the phage solution was thus demonstrated.

While 2 of the 7 control quarters no longer showed any *S. aureus* infection (28.6%) in the control examinations, 13 of 16 udder quarters in the experimental group were bacteriologically cured (BC) (81.3%). The difference in cure rates was significant (Fisher’s Exact Test, *p* = 0.026). A comparison of BC rates between experimental and control quarters revealed no significant difference between animals with a single infected quarter and those with multiple infected quarters (Z Test, *p* = 0.265).

**Figure 1:**
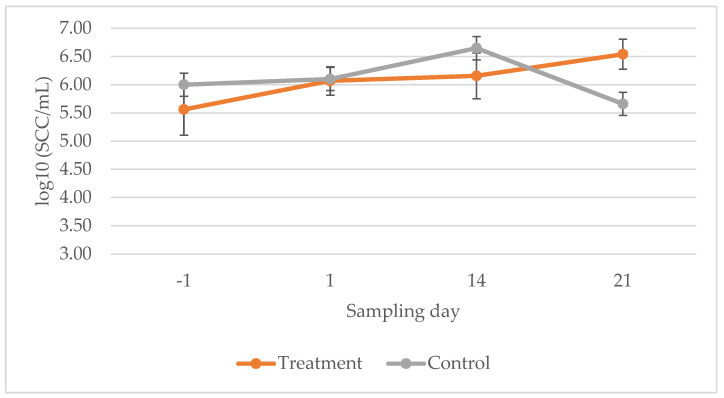
Change in the somatic cell count over the course of the study. Estimated mean values and 95% SEM (upper and lower bound) of the SCC are shown at the respective sampling date. Estimated with a generalised linear mixed model for the log_10_SCC (dots = estimated mean; error bars = standard error of the mean). No significant differences were found between the experimental and control groups, over time, or in the interaction between the group and the time point. −1 = Day before the start of treatment. 1 = First milking after the start of treatment. 14 and 21 = Control examinations on days 14 and 21.

The SCC (somatic cell count) did not differ significantly between the individual time points of the study, the assignment of the quarter to the experimental or control group, or the interaction of the two variables (*p* > 0.05). The SCC development during the experiment is shown in Figure 1.

When stratified by cure status, SCC development did not differ significantly between cured and non-cured quarters (Table 1), indicating that SCC alone was not predictive of bacteriological cure.

## 3. Discussion

Cure rates of *S. aureus* mastitis, whether spontaneous or antibiotic-induced, are typically low, particularly in subclinical or chronic infections. Given the low efficacy of conventional treatments and the growing problem of antimicrobial resistance, alternative therapies to antibiotics are required/increasingly being explored. Therefore, this pilot study investigated the tolerability and efficacy of a phage cocktail in an aqueous matrix when applied to healthy and *S. aureus*-infected udder quarters of dairy cows.

The results have shown that customized bacteriophage therapy can be both a tolerable and effective treatment. The bacteriological cure rate after bacteriophage application of 81.3% was 2.84 times higher than the spontaneous cure rate. This treatment effect exceeds those reported in most comparative antibiotic studies, where bacteriological cure rates for *S. aureus* mastitis typically range from less than 30% to about 50% [23,24,25]. Moreover, the efficacy observed in our study was also greater than that reported by Gill et al. (2006), who investigated the effect of intramammarily infused bacteriophage K in subclinically *S. aureus*-infected udder quarters of 24 lactating Holstein cows [5]. They compared the bacteriophage-treated quarters with quarters that were treated with a placebo (saline). The results showed that 16.7% (3/18) of bacteriophage-treated quarters achieved bacteriological cure, compared with 0% (0/20) in the placebo group. One possible explanation for the higher cure rates in our study is the use of a phage cocktail rather than a single phage in the study by Gill et al., which probably broadened the host spectrum and reduced the risk of bacterial escape. Furthermore, our previous *in vitro* experiments have shown that a very high phage dose is required to eliminate the *S. aureus* population in the mammary gland and in raw milk, as extensive non-specific binding between the phages and milk components must be assumed [21].

Due to the low bacteriological cure rates after antibiotic therapy, various studies recommend extending the duration of treatment in order to achieve higher bacteriological cure rates [25,26]. However, this inevitably leads to higher antibiotic consumption which in turn is detrimental to combating the development of antimicrobial resistance.

The scope and methodology of the study were specified by the corresponding animal testing authorization. In this respect, it was a pilot study. However, the most important confounders for the cure of intramammary *S. aureus* infections, like parity or high SCC in the last three milk controls, could already be balanced in this negative controlled study [27]. Although some animals had several infected udder quarters, the results do not indicate a significant effect of individual animals on outcomes.

The results obtained should be confirmed in a multicenter study with a larger sample size and more power. This is particularly important to ensure that the phage selection of the cocktail also covers the existing infections.

All mastitis therapy studies dealing with *S. aureus* run the risk of overestimating the bacteriological cure, as in many cases the *S. aureus* density in the milk is close to the detection limit of 100 CFU/mL [28]. By analysing the control and experimental quarters in parallel, at least the risk of non-identification was equal across groups. In addition, the definition of cure used here corresponds to that of antibiotic treatments. Longer longitudinal control studies are required to fully assess sustained bacteriological cure.

The application frequency and the interval between applications were chosen as a compromise between high application frequency and the increasing risk of iatrogenic infection with each additional application. The SCC results did not indicate that the treatment had any effect on inflammation in the udder quarters. This observation is consistent with the mechanism of action of bacteriophages. As with the use of antibiotics, bacteriophages are not able to directly influence the inflammation in the affected udder quarter. They can only destroy the target bacteria present in the quarter of the udder. Normalization of SCC usually takes weeks to months or never occurs after elimination of the pathogen, especially in chronic *S. aureus* infections [29]. The udder quarters examined showed high SCCs from the start of the experiment, and the 21-day follow-up period may have been insufficient to capture inflammatory resolution.

It is also possible that it is not the bacteriophages used but rather the lipopolysaccharide contamination, which is often part of the culture solutions during their reproduction, that is responsible for the inflammatory symptoms described in other articles.

Our investigations had revealed that the first batch of the bacteriophage solution was contaminated with lipopolysaccharides (LPS). Therefore, the treatments were carried out using newly produced, uncontaminated phage solutions. LPS-free production of bacteriophages is challenging, as LPS are present in various components of microbial culture media. The study found no evidence of insufficient tolerability when applying a phage mixture in aqueous solution to healthy and infected udder quarters.

One limitation of the study is the relatively small sample size. In addition, the efficacy evaluation was conducted on a single dairy farm. This could limit the generalizability of the results as farm-specific factors such as hygiene standards and microbiological environment could influence both the infection dynamics and the response to treatment.

In addition to demonstrating efficacy, this study highlights the potential of bacteriophage therapy as a promising, residue-free, targeted alternative to antibiotics, which is particularly important in the context of growing antimicrobial resistance. The specificity of phages also offers the possibility of precise treatment, although this requires prior identification of the bacterial strain and the production of suitable phage combination.

The results obtained should be confirmed in a multicenter study with a larger sample size. This is particularly important to ensure that the phage selection of the cocktail also covers the existing infections.

### Limitations

This study was conducted as a pilot trial and therefore has several inherent limitations. As already mentioned, the scope and methodology of the study were determined by the corresponding animal testing authority. The efficacy assessment was therefore carried out on a single dairy farm, and farm-specific factors such as management practices, pathogen ecology, or environmental conditions may have influenced the outcomes. Furthermore, the sample size was relatively small, which restricts statistical power and limits the precision of the estimated cure rates. This was nevertheless adequate for detecting clinically relevant treatment differences. These constraints underline the early-phase character of the work and highlight the need for confirmation in larger, multicenter studies.

## 4. Materials and Methods

### 4.1. Ethical Approval

This study was conducted as part of the project “Anwendung von Bakteriophagen in der Therapie von *Staphylococcus aureus* Mastitiden” (Application of bacteriophages in the therapy of *Staphylococcus aureus* mastitis, EIP agri) in accordance with the guidelines on good clinical practice (EMEA, 2000) [30] and in accordance with the “Guideline on the conduct of efficacy studies for intramammary products for use in cattle” (EMA, 2025) [31]. The trial registry number is 276 03 201 000 1358—LAVES Niedersachsen. The study complies with the Guidelines for Randomized Controlled Trials in Livestock and Food Safety (REFLECT checklist).

### 4.2. Farms and Animals

The study was conducted on two conventional German dairy farms in Lower Saxony (farm 1) and Saxony-Anhalt (farm 2) between October 2024 and January 2025. Farm 1 is a dairy farm near the microbiology laboratory with a herd of 62 dairy cows that are milked using a milking robot (Lely, Maassluis, The Netherlands). It is a small cubicle barn with high stalls and slatted floors. The animals are fed a partial total mixed ratio (TMR) and receive concentrated feed in the milking system. The output is 10,600 kg of energy-corrected milk (ECM) with a bulk milk somatic cell count of 180,000 cells/mL. Farm 2 is a dairy farm with 1015 dairy cows in Saxony-Anhalt. The animals are milked in a 2 × 14 herringbone milking parlor and kept in cubicle housing with deep stalls in performance groups and fed with full TMR. The lactation yield is 10,300 kg ECM with a bulk somatic cell count of 265,000 cells/mL.

### 4.3. Inclusion and Exclusion Criteria

Lactating cows with intramammary infection caused by *S. aureus* were eligible for inclusion. Infection status was confirmed by bacteriological culture of quarter milk samples obtained during prescreening of all dairy cows that had previously tested positive for *S. aureus* infections on the two farms. Quarter milk samples were collected immediately before regular mechanical milk removal.

Only cows that were clinically healthy apart from mastitis and not expected to enter the dry period during the study period were included. Cows of all parities and lactation stages were eligible. To avoid confounding treatment effects, cows that had received intramammary or systemic antimicrobial treatment within the preceding four weeks were excluded. Chronicity of infection was inferred from historical milk recording data, including persistently elevated somatic cell counts. All animals included in the study, except for one cow, had at least two milk tests > 100,000 cells/mL (German definition for chronic mastitis) in the last three milk tests prior to the start of the study, and except for two other cows, at least two milk tests > 200,000 cells/mL (international definition for chronic mastitis). In both the treatment and therapy groups, there were two cows that met the standard German definition for animals not eligible for therapy (3 consecutive measurements > 700,000 cells/mL).

Following prescreening, all *S. aureus* isolates identified on both farms were sent to Phage Technology Center GmbH (PTC, Bönen, Germany) to confirm *in vitro* efficacy of the previously developed phage combination [21] against the specific bacterial isolates present in the study population. The phage combination in an aqueous matrix was filled into suitable disposable syringes and brought to the microbiological laboratory in compliance with the cold chain and cooled there until use (<7 °C).

### 4.4. Study Design and Treatment

This study was designed as a two-stage, randomized, negative-controlled pilot trial. Farm 1 was used to assess tolerability and safety, while Farm 2 was used to evaluate therapeutic efficacy under field conditions.

For all treatments on both farms, teats were cleaned and disinfected prior to each infusion using standard aseptic procedures, including cleaning of the teat surface and disinfection of the teat apex with 70% ethanol. The bacteriophage preparation was administered using sterile, disposable syringes.

#### 4.4.1. Tolerability Assessment (Farm 1)

5 mL of the phage combination was injected by the veterinarians into the respective udder quarters 3 times at 12-h intervals. The animals were then monitored by the veterinarians for a period of 72 h, recording any clinical abnormalities.

#### 4.4.2. Efficacy Trial Design and Randomization (Farm 2)

On farm 2, udder quarters with confirmed *S. aureus* intramammary infection were enrolled in the efficacy trial. Infected quarters were randomly assigned to either the treatment group or an untreated control group in a ratio of 3:1 using a computer-generated randomization list. Laboratory Personnel performing bacteriological analyses were blinded to treatment allocation. The treatment and clinical examination were performed by different veterinarians.

#### 4.4.3. Treatment Protocol

Quarters allocated to the treatment group received intramammary administration of a bacteriophage cocktail. A volume of 5 mL was administered per quarter per application. Treatments were applied five times at 12-h intervals by a veterinarian. Quarters in the control group remained untreated.

#### 4.4.4. Sampling Timeline and Outcome Definitions

Control milk samples for bacteriological analysis and SCC determination were collected from all enrolled quarters 14 and 21 days after the last treatment. Primary outcome was BC of *S. aureus* infections at day 14 ± 3 and 21 ± 3. The absence of *S. aureus* cultured pre-treatment in both post-treatment samples at days 14 and 21 defined BC. A case was still defined as bacteriologically cured if a bacterial species other than the pathogen cultured pre-treatment was isolated in the post-treatment samples. If one post-treatment sample was contaminated, the outcome of the other post-treatment sample was used to ascertain the BC. SCC was evaluated as a secondary outcome to assess inflammatory response over time.

### 4.5. Microbiological Analyses/Laboratory Procedure

The microbiological analysis of the milk samples was conducted at the laboratory of the University of Applied Sciences and Arts Hannover (Germany) in accordance with the guidelines of the German Veterinary Association (GVA) [32]. The test tubes contained the preserving agent boric acid (Ly20) [33]. The somatic cell count was measured by flow cytometry with the SomaScope™ Smart (Delta Instruments B.V., Drachten, The Netherlands). For microbiological analysis, 10 µL of each milk sample was plated onto esculin blood agar (5% defibrinated sheep blood, Oxoid Deutschland GmbH, Wesel, Germany) and incubated aerobically at 37 °C for 48 h. The examinations were implemented after 24 h and after 48 h of aerobic incubation. An initial classification was performed by assessing the colonies formed according to their morphology and hemolysis patterns. They were then subjected to Gram staining and further biochemical tests (catalase activity (3% H_2_0_2_; Merck KGaA, Darmstadt, Germany), esculin hydrolysis). Gram-positive and catalase-positive cocci were categorized as staphylococci. Beta-hemolysis and the clumping factor-test (Staph Plus Kit, DiaMondiaL, Virotech Diagnostics GmbH, Vienna, Austria) were used to differentiate between non-*aureus* staphylococci (NaS) and *S. aureus*. *S. aureus* showed beta-hemolysis and was clumping factor-positive. All clumping factor-negative colonies were determined to be NaS. Milk samples were classified as *S. aureus* positive if ≥1 colonies were growing on the plates. Even if a milk sample was considered contaminated (>2 different pathogens were growing on the plate), all different colonies were examined to determine S. aureus positive samples. To verify bacterial genus and species, matrix-assisted laser desorption ionisation-time of flight (MALDI-TOF) was performed thereafter. All differentiated staphylococci were analyzed by MALDI-TOF (Microflex LT/SH smart, Bruker Daltonik, Bremen, Germany). A representative colony was used for MALDI-TOF analysis as described by Randall et al. (2015) [34].

The laboratory personnel performing the microbiological analysis was unaware of the origin of the milk samples.

### 4.6. Bacteriophage Mixture

Bacteriophages EB1.ST11, EB1.ST27, STA1.ST29 and STA1.ST107, isolated and provided by the PTC GmbH, Bönen, Germany, are sequenced lytic phages, which belong to the Class Caudoviricetes and are morphologically a myovirus (STA1) and a podovirus (EB1). A bacteriophage mixture was prepared for the trials as detailed in Titze et al. (2020) [21]. The total bacteriophage concentration of the preparation was set to 1 × 10^9^ PFU/mL.

The phage mixture was investigated for the LPS contents before it was used in the trials using a harmonised endotoxin test according to Ph.Eur. 11.6, 2.6.14:2024 (Pyrotell-T by the producer Associates of Cape Cod, Inc., East Falmouth, MA, USA). A maximum of 100 enzymatic units per milliliter (EU/mL) was defined as tolerable.

### 4.7. Statistics

The data were collected and analyzed using Excel, Office 2010 (Microsoft Corporation, Redmond, WA, USA), and SPSS (IBM SPSS 29.0.0.0, Armonk, NY, USA). The *S. aureus* IMI of an udder quarter represented the statistical unit. For every IMI, BC or no BC (encoded as 1 or 0, respectively) was determined according to the aforementioned definition, constituting the binary dichotomous-dependent variable. Treatment and control group are balanced in terms of stage of lactation (grouped: early, <101 days; mid, 101–200 days; late, >201 days; Fisher’s Exact Test *p* = 0.271), parity (grouped: ≤2, >2; Fisher’s Exact Test *p* = 0.345), SCC history of the last three milk controls (1 (low): all < 101,000 cells/mL, 2 (medium): not in 1 or 3, 3 (high): all > 700,000 cells/mL; Fisher’s Exact Test *p* = 0.858). Fisher’s Exact Test was used to compare treatment efficacy (BC) between the two groups. To assess the development of SCC during the trial, the target variable log10SCC was examined using a linear mixed model. The examination time point was a repeated measurement, and the treatment group and its interaction served as fixed factors. The assignment of a quarter in a cow was examined as a random variable. Statistical significance was assumed at α = 0.05.

## 5. Conclusions

To our knowledge, this is the first negative controlled *in vivo* study to evaluate the tolerability and efficacy of a *S. aureus* bacteriophage cocktail when applied locally to the mammary gland in cattle. Our results not only suggest that local intramammary treatment with a bacteriophage cocktail is tolerable but that it may also improve bacteriological cure compared to non-treatment of intramammary infections with *S. aureus*. The treatment of intramammary infections with bacteriophages has great potential as it is safe and free of antimicrobial residues. Further multicenter studies are required to confirm these findings and further assess the generalizability and long-term effects.

## Figures and Tables

**Table 1:** Somatic cell count (mean log_10_ cells/mL ± SD crude data) at baseline (−1) and follow-up stratified by treatment group and bacteriological cure status.

Sampling Day	−1 ^1^	1 ^2^	Cured	14 ^3^	21 ^3^
**Treatment**	5.56 ± 0.22 ^4^	6.07 ± 0.18	Yes	6.08 ± 1.23	6.63 ± 0.27
			No	5.86 ± 0.80	6.50 ± 0.45
**Control**	6.00 ± 0.46	6.10 ± 0.25	Yes	7.18 ± 0.16	7.00 ± 0.01
			No	6.02 ± 0.33	5.66 ± 0.01

^1^ Day before the start of treatment, ^2^ First milking after the start of treatment, ^3^ 14 and 21 = Control examinations on days 14 and 21, ^4^ mean ± standard deviation of log_10_-transformed somatic cell counts.

## Data Availability

The original contributions presented in this study are included in the article. Further inquiries can be directed to the corresponding authors.
